# Can "presumed consent" justify the duty to treat infectious diseases? An analysis

**DOI:** 10.1186/1471-2334-8-29

**Published:** 2008-03-06

**Authors:** Murat Civaner, Berna Arda

**Affiliations:** 1 Uludag University School of Medicine, Department of Medical Ethics, Bursa, Turkey; 2 Ankara University School of Medicine, Department of Medical Ethics, Ankara, Turkey

## Abstract

**Background:**

AIDS, SARS, and the recent epidemics of the avian-flu have all served to remind us the debate over the limits of the moral duty to care. It is important to first consider the question of whether or not the "duty to treat" might be subject to contextual constraints. The purpose of this study was to investigate the opinions and beliefs held by both physicians and dentists regarding the occupational risks of infectious diseases, and to analyze the argument that the notion of "presumed consent" on the part of professionals may be grounds for supporting the duty to treat.

**Methods:**

For this cross-sectional survey, the study population was selected from among physicians and dentists in Ankara. All of the 373 participants were given a self-administered questionnaire.

**Results:**

In total, 79.6% of the participants said that they either had some degree of knowledge about the risks when they chose their profession or that they learned of the risks later during their education and training. Of the participants, 5.2% said that they would not have chosen this profession if they had been informed of the risks. It was found that 57% of the participants believed that there is a standard level of risk, and 52% of the participants stated that certain diseases would exceed the level of acceptable risk unless specific protective measures were implemented.

**Conclusion:**

If we use the presumed consent argument to establish the duty of the HCW to provide care, we are confronted with problems ranging over the difficulty of choosing a profession autonomously, the constant level of uncertainty present in the medical profession, the near-impossibility of being able to evaluate retrospectively whether every individual was informed, and the seemingly inescapable problem that this practice would legitimize, and perhaps even foster, discrimination against patients with certain diseases. Our findings suggest that another problem can be added to the list: one-fifth of the participants in this study either lacked adequate knowledge of the occupational risks when they chose the medical profession or were not sufficiently informed of these risks during their faculty education and training. Furthermore, in terms of the moral duty to provide care, it seems that most HCWs are more concerned about the availability of protective measures than about whether they had been informed of a particular risk beforehand. For all these reasons, the presumed consent argument is not persuasive enough, and cannot be used to justify the duty to provide care. It is therefore more useful to emphasize justifications other than presumed consent when defining the duty of HCWs to provide care, such as the social contract between society and the medical profession and the fact that HCWs have a greater ability to provide medical aid.

## Background

### Risks and attitudes

In the course of providing health care service, health care workers (HCWs) are continually exposed to many work-related health risks. One of these risks is the exposure to infectious diseases. These diseases can include the flu, AIDS, tuberculosis, and hepatitis, and can be transmitted through physical contact, exposure to contaminated blood, or via the respiratory system. And, needless to say, such risks do indeed at times prove fatal. The consequences of occupational exposure to pathogens are not limited solely to bodily infections. Each year, thousands of HCWs are adversely affected by psychological trauma stemming from months of anxiously awaiting the results of serological tests, tests made necessary due to potential infection incidents. The anxiety experienced by HCWs is related to the perception of risk from the incident and the resulting infection that may occur, and by the worry of what the reactions of others might be, such as colleagues, family, and friends, all who have to be informed. During this uncertain waiting period HCWs will frequently experience intrusive thoughts, problems concentrating, difficulty sleeping, frequent loss of temper, and a decrease in sexual desire, which can act as a catalyst to exacerbate any pre-existing and unresolved emotional issues [1]. And if it turns out that the health care worker has indeed been infected by one of these contagions, the serious personal consequences to that health care worker can include the postponement of childbearing, damaged personal relationships, having to alter sexual practices, experiencing the side effects of prophylactic drugs, chronic disabilities, loss of employment, denial of worker compensation claims, possible need for a liver transplant, and premature death [2].

AIDS, SARS, and the recent epidemics of the avian-flu have all served to remind us of the occupational risks faced everyday by HCWs; the result being that the recent appearance of these diseases has forced this issue onto the common agenda and helped to spark renewed interest in the debate over the limits of the moral duty to treat. In seeking an answer to this question it is useful to have an understanding of the occupational risks faced by HCWs as well as an understanding of the attitudes of HCWs to these risks. For example, studies conducted in various countries have shown that, especially when there was a risk of being infected with AIDS, HCWs may refuse to treat a patient on the grounds that there is a risk of being infected by this patient [3-11]. And despite the fact that the hepatitis viruses are transmitted more easily than HIV, it is the fear of being infected with HIV that causes many HCWs to experience the greatest amount of stress and anxiety [12]. In a study which compared the relative risks of transmission of both HBV and HIV, the reasons for physicians' underlying fears of particular contagions were also investigated and described. [13]. According to the study, people initially percieve the risk to be greater when there is a high likelihood of death involved with infection (as with HIV) even though there may be less risk of infection, as opposed to when there is a higher risk of infection but a lower risk of death involved with that infection (as with HBV). Additionally, since the likelihood of sexually transmitting HBV between heterosexual partners is less than that of transmitting HIV, the consequences of HBV infection are again percieved to be less severe than the consequences of HIV infection. In this way, the hazards posed by HBV infection conflict less with the obligation to protect family members from harm. It was also found to be important that there is less of a stigma attached to having HBV than there is to having HIV. And, finally, the fact that there is a vaccine for HBV infection, which is more than 90 percent effective (for vaccinated HCWs the risk of death from infection is reduced by a factor of nearly twenty), also was found to greatly influence the perceptions of the physicians.

Additionally, factors other than a fear of the contagion can contribute to the reluctance to treat a particular patient. Some physicians and dentists express concern that if it is discovered that they treat patients with AIDS, then those patients who don't have HIV may shun their practice. Still, other physicians insist they do not know enough about HIV infection and are too busy to learn [14]. Another reason for which a physician may refuse to treat HIV-positive patients is that the physician feels they have a duty to protect their other patients, basing their reasoning on the principle of "First do not harm". By treating HIV-positive patients they claim that they may potentially be putting their other patients at risk for infection [15]. Furthermore, as has been reported, there is always the possibility that when a HCW is able to reject the patient based on a more benign excuse, for example if the patient does not have enough money, it is even easier, and all the more likely, for treatment to be refused, even though this refusal was done in the interest of protecting the physical health of the individual health care provider [14].

### Theoretical framework for the duty to treat

In the literature, most studies have concentrated primarily on the attitudes and rationale behind the refusal to treat. Before one can set out to effectively explore the attitudes of HCWs however, it is important to first consider the question of whether or not the "duty to treat" might be subject to contextual constraints, such as providing health care to a patient suffering from an infectious disease which may be particularly contagious or for which adequate treatment measures may not yet be available.

Clark, in his article about physicians' duty to treat, claimed that there are three reasons in which such a duty is grounded [16]:

"... since the ability to render aid is greater, the obligation to assist is (...) elevated. Second, by consideration of Daniels' argument that by freely joining a profession designed to combat disease, one consents to some standard of risk, and third, by realizing that the profession has flourished due to socially negotiated promises to be available in such times of duress."

In his article "Duty to treat or right to refuse", Daniels argues that when a person chooses a career in a particular profession, it must be understood by all parties that this individual has both accepted and is willing to take the risks that are inherent to that profession [14]:

"Consent is crucial where obligations to take risks exist in various occupations or professions. For example, we assume that in choosing their careers, undergoing the training involved, and agreeing to follow the codes and practices regulating their work, firefighters and police have given consent to facing the significant risks they are obliged to take. There are strong parallels to medicine. People who enter medical fields clearly had alternatives. There is a general understanding that physicians face an increased risk of contagion from disease, an understanding refined during schooling and training."

Daniels proposes, however, that some situations can exceed the standard level of risk (SLR) [14]:

"For example, it is common to screen new house staff and nurses in medical centers to determine whether any individuals face special risks of contagion, such as immunosuppression or pregnancy. Those at high risk may then be asked to avoid certain treatment situations, materials, or hospital areas.(...) Protecting immunosuppressed providers is reasonable "risk management", a measure taken to reduce bad outcomes. But such special protection supports the claim that only standard risks are included in the duty to treat.(...) Some nosocomial risks clearly take us beyond what duty requires."

It is perhaps more illustrative if this argument (from this point on, this statement will simply be referred to as the "presumed consent") is written in classic form:

#### Premise 1

Health care services should be provided to patients who have a contagious disease.

#### Premise 2

Contracting an infectious disease while providing health care services to a patient with a contagious disease is an occupational risk.

#### Premise 3

It is generally assumed that by joining the health care profession physicians have given their consent to be exposed to an increased risk of disease contagion. This assumption is based on the following facts:

a. There is a general understanding that physicians face an increased risk of contagion from disease, an understanding refined during schooling and training.

b. People who enter medical fields clearly had alternatives.

#### Premise 4

Some nosocomial risks clearly take us beyond what duty requires.

#### Conclusion

There is a moral duty to treat patients who have a contagious disease so long as the risk to the HCW is below the SLR.

If we are to accept this argument, then the pressing question becomes how to determine and define the risks which are deemed to be standard and acceptable versus those which are believed to exceed and, indeed, outweigh the duty of the health care provider to treat. In order to begin to answer this question, it will be useful to investigate the nature of the choice (and all that goes along with making it) that an individual makes when they decide to enter a particular profession. For instance, how wise is it to assume that at the time of choosing their future profession the HCW was fully aware of the risks involved with such work? Perhaps they were not made aware of the risks until their education and training. Furthermore, if they were aware of, and fully appreciated, the risks prior to deciding on a particular profession, would they have even chosen that profession in the first place? And, finally, how is the SLR to be determined, and which of the infectious diseases would then exceed this SLR? In order to effectively analyze the presumed consent argument it is necessary to have an awareness of the diverse opinions and beliefs of HCWs and, also, to understand their different motives and backgrounds. Additionally, knowledge of what HCWs feel about the risk concept and of how they feel about their duty to treat patients with contagious diseases can also be of great value to educators as they plan their curricula and it can be used by the authorities in charge of health care systems in order to better organize their services. The purpose of this study was to analyze whether or not the third premise grounds the duty to treat, namely, "it is generally assumed that by joining the health care profession physicians have given their consent to be exposed to an increased risk of disease contagion". In order to carry out this analysis, the opinions and beliefs of physicians and dentists regarding the occupational risks of infectious diseases were investigated; and, by extension, the argument that the notion of "presumed consent" may be grounds for supporting the HCWs' duty to treat was also analyzed.

## Methods

For this cross-sectional survey, the study population was selected from among physicians and dentists in Ankara, the capital of Turkey. A self-administered questionnaire designed to assess the beliefs and opinions of the participants regarding the occupational risks of infectious diseases was used. This questionnaire was also used to obtain the socio-demographic information of the participants. The 17 items on the questionnaire were developed by reviewing previous studies in the literature [1,3-10]. A draft of the questionnaire was distributed to experienced health care professionals and later revised based on their criticism and suggestions.

In both of the universities in which this study was conducted there are ethics committees which had been established for the purpose of determining the ethical appropriateness of pharmaceutical trials using humans; since our study involved only the use of a questionnaire and not an experimental drug, we did not apply for approval from either of these ethics committees. Instead, written permission to carry out the study was granted by the dean of the faculty of medicine and by the chief manager of university hospitals. In addition, all of the potential participants were fully informed about the aim and structure of the study. Furthermore, potential volunteers were all made aware that participation was strictly voluntary and that all of the answers they provide would be done so anonymously.

The questionnaire was administered to a total of 373 health care workers: all of the 236 physicians who work in surgical specialties at the Ankara University Ibn-i Sina Hospital and to all of the 137 dentists in the Gazi University Faculty of Dentistry. Dentists were included in this study because, aside from being HCWs themselves, there are a number of studies in the literature which show that dentists, citing various reasons, may also refuse to treat patients with contagious diseases. And, in order to better assess the fact on which the third premise of presumed consent is based, we decided to include only professional health care workers, instead of students and others who might still be in the process of deciding whether or not to currently enter the field.

In total there were 230 participants, 101 physicians and 129 dentists, who completed the questionnaire, for an overall response rate of 61.7%. The questionnaire was later sent back to the non-respondents one month after the first survey, and 28 of these were completed and returned to us. The mean age of the participants was 33.8 ± 9.6 years, while 56.5% were male and 43.5% were female. Additionally, the average amount of time that they had been working in the medical profession was found to be 8.5 years (min. 0, max. 40). All of the data was collected anonymously. The difference between the two groups, physicians and dentists, was compared using the chi-square test, with a p-value of <0.05 accepted as statistically significant. All analyses were carried out using SPSS 11.5.

## Results

Of the HCWs surveyed in this study, roughly half stated that they understood that by choosing their profession they would be exposing themselves to an increased risk of contracting contagious disease (55.2%). And at the time of entering the faculty, 24.4% of the participants expressed that they were unaware of any increased risks; however, they later learned of these risks during their education and training. In other words, 79.6% of the participants stated that they had known about the risks either at the time they chose their profession or that they had later learned of the risks during their training and education. Additionally, 6.5% of the participants answered that they had only come to realize the kinds of risks they would face after starting to work. The percentage of participants who claimed that if they had been aware of the risks earlier they would not have chosen to enter or continue in the medical profession was 5.2%.

Listed in Table 1 are statements which physicians and dentists chose as best reflecting their personal opinions regarding the occupational risks of infectious disease. In general, the physicians, prior to their education and training, were significantly more aware of the potential risks associated with their profession than were the dentists (p < 0.05). A significantly higher percentage of the dentists however, stated that they only learned of the occupational risks of dentistry during their education and training (p < 0.05). There was no statistically significant difference between the two groups in terms of the other opinions questioned.

**Table 1:** Opinions of the participants regarding the occupational risks of infectious diseases (%)

		**Physicians ***(n:101)*	**Dentists ***(n:129)*
1	When I entered the faculty I knew that I would be at an increased risk for exposure to infectious diseases because of my chosen profession.	63.4*	48.8
2	I might not have chosen this profession, if these risks had been thoroughly explained to me before I entered the faculty.	1.0	4.7
3	I did not know that I would be at an increased risk for exposure to infectious disease when I entered the faculty. However, I later learned of these risks during my education and training.	16.8	30.2*
4	I might not have chosen this profession, if these risks had been thoroughly explained to me during my education and training.	1.0	3.1
5	I only came to understand what kind of risks I would be exposed to when I started to work after graduation.	7.9	5.4

* p < 0.05

The participants were also asked whether or not they agree with the argument "When people choose, and continue to practice, the medical or dentistry profession, they are then required to accept all of the occupational risks resulting from the infectious diseases they might confront". The aim of this question was to determine whether or not the HCWs each have their own individual working-definition for the SLR. Of the participants, 57.4% believed that there is such a level. 52.2% felt that certain diseases would exceed the level of acceptable risk unless specific protective measures were implemented, and 5.2% said that some diseases were always beyond the SLR, no matter what precautions might be taken. No statistically significant difference was found between the physicians and the dentists.

Listed in Table 2 are the diseases which, under certain circumstances, were cited as potentially exceeding the SLR. Among the participants who stated that there would be a SLR for providing health care to the patients of specific diseases unless protective measures were implemented, AIDS and Hepatitis C and B were the most frequently cited of these diseases (71.7%, 64.2%, and 56.7%, respectively). The participants who felt that some diseases would always exceed a SLR expressed, that Hepatitis B, Tuberculosis, and Bacterial meningitis always would go beyond the SLR (41.7%, same for all). According to these participants, the occupational risk of potentially being infected with HIV is paramount to all other risks. Percentage-wise, AIDS was the most frequently mentioned disease that would exceed the SLR, more so than SARS.

**Table 2:** The diseases regarded as exceeding the SLR (%)

	**If there are no protective measures available, some diseases below would exceed the SLR ***(n:120)*	**Some diseases below always exceed the SLR ***(n:12)*
AIDS	71.7	75.0
Hepatitis C	64.2	58.3
Hepatitis B	56.7	41.7
SARS	52.5	66.7
Tuberculosis	35.0	41.7
Bacterial meningitis	34.2	41.7

All of the participants who answered that some diseases would be beyond the SLR were then asked what criteria they used to make their determination. The most commonly expressed criteria, in order, regarding the diseases, were the likelihood of transmission, whether or not protective measures are available, and whether or not immunization is possible (66.7%, 65.2%, and 58.3%, respectively). The distribution of these criteria among the physicians and dentists can be seen in Table 3. Physicians expressed significantly more often than dentists that if there was no immunization or treatment available for a particular disease, then that disease would exceed the SLR (p < 0.01). In terms of other criteria, there were no significant differences observed between the two groups.

**Table 3:** Frequency of criteria used to determine whether a particular disease is below the SLR

	**Physicians ***(n:55)*	**Dentists ***(n:77)*
Whether specific immunization is available	72.7*	48.1
Whether protective measures are available	69.1	62.3
Probability of transmission	63.6	68.8
Probability of cure	54.5*	29.9
The mechanism of disease transmission	49.1	49.4
The mortality rate of the disease	38.2	46.8
Prevalence of the disease in the general population	25.5	22.1
The social (stigmatic and discriminatory) impact of the disease	14.5	14.3

* p < 0.01

## Discussion

The primary aim of this paper is to evaluate the claim that presumed consent may constitute grounds for the moral duty to treat. The presumed consent argument is valid, because its conclusion should logically be accepted if its premises are taken into account. To analyze the soundness of the argument we carried out a survey investigating the opinions of HCWs about the occupational risks of infectious diseases. In total, 79.6% of the participants said that they either had some degree of knowledge about the risks when they chose their profession or that they learned of the risks later during their education and training. In other words, one fifth of the participants either lacked adequate knowledge about the occupational risks when they chose their profession or were not sufficiently informed of these risks during their faculty education and training. This means that the assumption stated in Premise 3 may be wrong for an important proportion of health care workers. It seems reasonable to suggest that the words "there is a general understanding" would be misleading if used to characterize a social concept of which the applicability and, indeed, the very existence, are yet to be established by sociological studies.

It is also useful to discuss the other problems associated with presumed consent; in particular, the difficulty of choosing a profession autonomously, the constant level of uncertainty present in the medical profession, the near-impossibility of being able to evaluate, in retrospect, whether or not every individual was informed, and the seemingly inescapable problem that this practice would legitimize, and perhaps even foster, discrimination against patients with certain diseases.

### Requisites for the presumed consent argument

If we are to use the presumed consent argument, then the findings of this study indicate that when a new epidemic of a contagious disease occurs, or when the medical profession is confronted with a disease for which no immunization or treatment options are available, some HCWs are not bound by the duty to treat according to the presumed consent argument. This seems potentially problematic and demands serious consideration. How appropriate is it to describe the healthcare provider's responsibility and duty as stemming from their 'consent'? To address this question, it is helpful to reflect on the conditions required for an individual to be able to give consent that is well-informed.

For an HCW's consent to be informed, the following should first be explained to them: (a) the risk posed by each of the contagious diseases known at that given time, (b) commonly agreed criteria and definitions of situations that would surpass the SLR, and (c) the fact that there will always be a degree of uncertainty involved with working in the medical profession, as new risks may emerge at any point during one's professional life. If not necessarily when they choose their profession, then at least after being given the relevant knowledge during education and training, the person's choice should be regarded as informed. It should therefore be ensured that HCWs are acquainted with each new and emerging risk, and with any methods of prevention developed during or after their education and training. If a person's choice is to be confidently regarded as informed, it is imperative that these conditions be met. Of course, the question now becomes: how possible is it to satisfy all these conditions?

### Choosing a profession: how autonomous can it be?

How autonomous is an individual's choice of profession? It is quite easy to imagine more than one answer to this question, but one thing is for sure: any thoughtful answer would acknowledge that choice is determined both by factors that are under the control of the individual and by factors that are not. Personal factors such as educational status, perception of the world and ambitions all influence an individual's choice of profession strongly. Nevertheless, factors outside the individual's control also play a large role in determining that choice. The environment in which the person grew up – their family life, the jobs of their parents, their community, social class and culture – all contribute to forming that individual's background, which (needless to say) has a very large influence on the opportunities and choices available to them.

Even though a person may not have been sufficiently informed when they chose their profession, it can be argued that during their education and training they will learn all relevant knowledge about the occupational risks associated with working in the medical profession. If so, it is fair to assume that when this individual begins to work after graduation they will be willing to confront any of those risks. In theory at least, it can be presumed that every student who passes their exams and goes on to graduate from the faculty is informed of the risks; so it can be argued that all HCWs who are currently active in their profession have consented to accept the risks posed by all the contagious diseases known at the time of their graduation. Of course, the diverse factors that determine the quality of education, such as the particular educational methods used, the course content, the abilities and knowledge of the instructors, role-models, and the personal features and motivations of students, are all potential sources of variation. But for the sake of argument, let us assume that a standardized education program is implemented throughout all medical schools. If that were the situation, then the argument that the individual has been made aware of the occupational risks during education would be true to the extent that the education program addressed those risks sufficiently. Nevertheless, it would be hard to claim that presumed consent is valid for every individual. For many people, a degree in medicine is very costly, both financially and in terms of time and energy. Under such circumstances, it is difficult for a person to quit their schooling despite the awareness they gain of the occupational risks involved. Individuals might feel pressured and confused by the two options confronting them: on the one hand, dropping out of medicine and forfeiting all the time, effort and money spent on schooling towards that aim; and on the other, reluctantly accepting the occupational risks, which may look frightening to the individual at that moment. Of course, it is important to remember that the person chose the medical profession in the first place, and numerous positive and beneficial elements are associated with working within it, which may ultimately serve to temper and override the individual's fear of the risks. An additional source of pressure may be that, for whatever reasons, switching educational tracks is too difficult; it may appear too daunting or be financially untenable. It seems likely that in the end the individual will choose to continue with their education and embark on a career in medicine, despite hesitation and fear of the risks. It is difficult to describe a decision made under conditions of such uncertainty and stress as 'informed'.

As can be seen, we do not choose our profession from among a wide array of possibilities spread out in front of us by thoroughly researching each one so that we are fully informed of its nature; everybody's options are different and it is a large and difficult task to make oneself sufficiently informed of them. Moreover, as described above, the decision to quit medical school can be quite difficult: on one side of the dilemma there are occupational risks that must be accepted regardless of misgivings on the part of the individual; on the other side, very influential factors pressure the individual to continue their medical education. Thus, the claim that "People who enter medical fields clearly had alternatives" is debatable and sometimes even doubtful. In theory it sounds right; nobody has to be a physician. But in practice, having alternatives does not mean that all our decisions are made freely or autonomously.

### Uncertainty in medical professions

Theoretically, the fundamental problem with the presumed consent argument is that it cannot explain why there is always some degree of uncertainty about the occupational risks of working in the medical profession, particularly stemming from new and emerging diseases. Quite simply, if there was little or no knowledge of a risk at the time the individual became informed and gave their (tacit) consent, then this individual never accepted the risk, implicitly or otherwise, because it was unknown at the time the individual was informed. From a historical perspective, it is possible to see that while the medical profession was once concerned only with treating diseases, its vocational responsibility came in time to include preventative, promotive and rehabilitative healthcare services. As the notions of human rights and patient rights have developed and become widespread, perceptions about the health profession have changed at the community level. Diseases that once killed millions of people can now be treated with a simple medicament, but today we are faced with new and challenging diseases unheard of in the past. As a result, the continuous cycle of change – spurred on by greater knowledge and technological advances and confrontations with new and untreatable diseases – serves to alter the identity and nature of the medical profession, and out of all this arises a constant degree of uncertainty. This characteristic uncertainty is present both when the individual chooses the profession and throughout their education and training period – and, indeed, for the entirety of their professional career. It is therefore not possible for someone to be fully informed when they choose their profession, nor is it possible for them to become fully informed during their education and training; nevertheless, the person should be informed about the uncertainty involved with working in the medical profession. In the light of this uncertainty, answers such as "if I had known I would not have chosen it" are not very meaningful, because there is no way to anticipate all the potential risks one might encounter in the course of a professional life. The only sure thing amid the uncertainty is that diseases such as SARS and avian 'flu will always continue to emerge.

### Practical concerns

So far in the discussion, the difficulties of satisfying the conditions needed to validate the presumed consent argument have been described. It seems virtually impossible to fulfill all these conditions satisfactorily. At this point it is important to discuss two particular problems regarding the argument itself.

First, it seems nearly impossible to evaluate individually whether the choice made by every HCW to enter the medical profession was informed. The only way to do that would be laboriously to ask each HCW whether they were informed when they decided on the health care profession. Even then, irrespective of whether the person's answer to this question reflects the truth, the only thing that could be learned from such a broad and extensive interrogation would be the individual's perception, not their actual knowledge. By extension, we could not question the participants' level of knowledge in this study, but rather how they perceive their level of knowledge. Because it is futile to seek objectivity in people's perceptions, it would not be sound to use those perceptions to determine whether the HCW's choice was informed, and thus whether they have taken on the duty to treat. And if these perceptions are regarded as subjective, as they should be, then it would be very difficult to develop a set of standard criteria that could be used to establish whether a HCW has been informed. This would also complicate efforts to reach a consensus on forming criteria by which various levels of risk could be defined universally. Unfortunately, such difficulties can only hamper efforts to protect the right of every patient to receive the best treatment available.

The second problem is that there is very likely to be more discrimination against patients with certain diseases, as HCWs use this argument to justify their refusal to treat those diseases. The World Medical Association's Declaration of Geneva, which states the basic moral values of the medical profession, specifies that there should be no discrimination, regardless of the circumstances: "I will not permit considerations of age, disease or disability, creed, ethnic origin, gender, nationality, political affiliation, race, sexual orientation, or social standing to intervene between my duty and my patient" [17]. However, the physicians' right to choose their patients is described by the same organization in another policy called "Twelve Principles of Provision of Health Care in any National Health Care System" [18]: "Any health care system should allow the patient to consult the physician of his choice, and the physician to treat only patients of his choice, without the rights of either being affected in any way." For regulations at the national level, it is generally held that if there is an urgent need for medical care [19,20], or if no other physician is available to whom the patient can apply [21,22], this right would be negated and the available physician must treat the patient. If we look at these obligations in reverse, we can see that the physician might refuse to provide health services to the patient if there is no urgent need for medical care and/or if another physician is around to whom the patient can be referred. Besides, the physician might also refuse the patient on the grounds that their prejudices may adversely affect the advice or treatment that they provide [20]. In actuality, this flexibility was written into the regulations in order to ensure that the patient receives the highest quality care possible; but as can be seen, it could also be abused. And if the presence or absence of consent is used as a criterion to define the duty to treat, in addition the existing flexibilities, then a mechanism would be created by which HCWs may freely, and perhaps excessively, discriminate among patients.

To summarize, considering all the points discussed above, the soundness of the presumed consent argument can be doubted. Therefore, it should not be claimed that there is a duty to treat on the basis of the presumed consent argument, as the argument itself is not persuasive.

It is also important to note that 5.2% of our participants said that they would not have chosen this profession if they had been informed of the risks. In other words, most of those HCWs who claimed that they were uninformed of the occupational risks when they entered the faculty, or were not fully informed of them during their education period, stated that they still would have chosen the medical profession even if they had been more aware of the risks. This finding tells us that, generally speaking, HCWs place relatively little importance on being informed beforehand. Further support for these findings comes from the answers given by the participants to the other questions. Nearly half said that there is no SLR, and the other half felt that the diseases they evaluate would not surpass SLR if the appropriate protective measures are available. Also, Table 2 indicates that at least 28.3% of the participants thought that none of the diseases listed in that table would exceed the SLR regardless of circumstances. It can therefore be concluded that a large majority of the HCWs place more emphasis on their working conditions than on being informed beforehand. In addition, the criteria most commonly stated by the participants for determining the SLR were the likelihood of transmission of a disease, whether protective measures are available and whether immunization is possible. Each of these criteria is related to protecting the HCW from infection, not to the treatment or the effects of a particular disease. This means that as long as protective measures are available, the HCWs would regard a given disease as below the SLR, so it has nothing to do with being informed beforehand. Besides, the only disease used as an example in this study that could be claimed to exceed the SLR was SARS; AIDS, hepatitis C, hepatitis B, tuberculosis and bacterial meningitis all fell below the SLR according to these criteria. Nevertheless, only 30.9% of the participants suggested that SARS would surpass the SLR. To put this into perspective, SARS was not even observed until 2003, and the research in the present study was conducted in 2004 and 2005. Therefore, most participants in this study were not aware of SARS when they chose the medical profession, nor were they ever informed of it during education or training. They nonetheless felt that the duty to treat pertained even to patients with SARS. All of this suggests that factors more useful and relevant than presumed consent influence the decision of HCWs to choose and continue in the medical profession; these factors may include the social contract between society and the medical profession, and the greater ability of HCWs to provide medical care [16]. It is these factors that should be investigated and emphasized when defining a moral duty to treat.

This study could be limited by several factors. The first limitation could be due to a socially desirable response bias; some participants might have given what they perceived as the 'right' answers to the questions rather than the answers that reflect their opinion or belief. In order to address this concern, future studies could benefit by using qualitative methods, which provide more reliable results about the motives and opinions of participants. Also, this study was not prospective, so recall bias might have affected the responses of the participants. Furthermore, the extent to which the results of this study are applicable to HCWs such as nurses or physicians who work in internal specialties is uncertain. Future studies that include other HCWs as participants may broaden our understanding of the beliefs and opinions of HCWs, thereby allowing us to state our claims and shape our arguments more precisely. Finally, it should be mentioned that the response rate for this study (61.7%) was slightly lower than is generally expected for a survey. Nevertheless, despite all these methodological limitations, we believe that our findings support our conclusion about the persuasiveness of the presumed consent argument.

## Conclusion

If we use the presumed consent argument to establish the duty of the HCW to provide care, we are confronted with problems ranging over the difficulty of choosing a profession autonomously, the constant level of uncertainty present in the medical profession, the near-impossibility of being able to evaluate retrospectively whether every individual was informed, and the seemingly inescapable problem that this practice would legitimize, and perhaps even foster, discrimination against patients with certain diseases. Our findings suggest that another problem can be added to the list: one-fifth of the participants in this study either lacked adequate knowledge of the occupational risks when they chose the medical profession or were not sufficiently informed of these risks during their faculty education and training. As we stated above, in order for a candidate HCW to be informed literally, three items should be explained to them: (a) the risk posed by each of the contagious diseases known at that given time, (b) commonly agreed criteria and definitions of situations that would surpass the SLR, and (c) the fact that there will always be a degree of uncertainty involved with working in the medical profession, as new risks may emerge at any point during one's professional life. In this study it has been shown that at least some HCWs may not be informed of (a). Also, it is not currently possible to inform HCWs of (b) since there are no widely-agreed criteria and definitions to allow for a universally accepted SLR; and there is currently no standard education for all HCWs to ensure that (c) is satisfied. Considering this in addition to the problems mentioned above, the third premise of the presumed consent argument appears implausible and, consequently, the duty to treat cannot be grounded persuasively on the consent assumption. It is therefore more useful to emphasize justifications other than presumed consent when defining the duty of HCWs to provide care, such as the social contract between society and the medical profession and the fact that HCWs have a greater ability to provide medical aid.

Furthermore, in terms of the moral duty to provide care, it seems that most HCWs are more concerned about the availability of protective measures than about whether they had been informed of a particular risk beforehand. It seems important that further research be carried out to improve understanding of the opinions and perceptions of HCWs and the basis of their definitions, as this information could prove very helpful in defining a duty to treat that can be effectively put into practice. It is also important that a well-organized ongoing educational program that is needs-based and easily accessible be provided to HCWs at both the graduate and postgraduate levels. In particular, this program must be continuously updated regarding AIDS and other diseases that may cause the HCWs to behave discriminatively towards patients, even though these diseases are below the SLR. Such continuing medical education is the best answer to the justification "When I chose the profession/when I graduated, this disease did not exist!" for refusing treatment. Emphasizing the social role of HCWs, and educating them about the professional obligations derived from the social contract betweeen the profession and the wider social order, would further reduce that kind of reasoning. In addition, stricter standards for the duty to provide care should established by determining the criteria for a SLR and identifying the situations and conditions that would exceed this SLR. Each of these measures could serve to remind HCWs that they have a moral responsibility, as individual HCWs, to be aware of professional obligations and to act as responsible members of the profession. Moreover, the working environment of HCWs should be provided with preventative measures that can be applied both generally and specifically and should emphasize their use. For a circumstance in which a preventative measure has been developed for a disease but is not available for treating a particular case, it would not be easy to justify the claim that there is an undeniable duty to provide care at that moment.

As Dr. Singer says: "There is a threshold beyond which health care workers aren't obliged to take personal risks. We don't expect firefighters to jump into a burning pit, or police officers to throw themselves in front of a bullet. How health care workers define this threshold is an intensely personal decision. ... But obviously, it has serious implications for our collective response to a problem like SARS." [23]. It is clear that to rely upon the presumed consent argument to define the duty to treat will not make our collective response to potential epidemics such as SARS or avian 'flu any more effective or robust.

## Abbreviations

HCWs: Health care workers; SLR: Standard Level of Risk

## Competing interests

The author(s) declare that they have no competing interests.

## Authors' contributions

MC and BA have contributed equally to the conception, design and writing of the manuscript. All authors read and approved the final manuscript.

## Appendix

The questions referred to:

• Whether the participant knew when they chose their profession that they would have an increased risk of being infected by a contagious disease?

• If the participant did not know of this occupational risk, whether or not they later learned of it during their training?

• If they still have not been formally made aware of the risks, do they think that they have enough knowledge about the current risks that they face?

• If they had known about the risks earlier, would they still have chosen this particular profession?

• When somebody chooses to be a physician or a dentist, are they obligated to accept all of the occupational risks regarding infectious diseases?

• If not, what criteria must we use to determine that a particular disease is below the SLR?

• Which diseases are below the SLR?

## Pre-publication history

The pre-publication history for this paper can be accessed here:


